# Visualization of Cortical Projection Neurons with Retrograde TET-Off Lentiviral Vector

**DOI:** 10.1371/journal.pone.0046157

**Published:** 2012-10-05

**Authors:** Akiya Watakabe, Shigeki Kato, Kazuto Kobayashi, Masafumi Takaji, Yuki Nakagami, Osamu Sadakane, Masanari Ohtsuka, Hiroyuki Hioki, Takeshi Kaneko, Hiroyuki Okuno, Takashi Kawashima, Haruhiko Bito, Yoshihiro Kitamura, Tetsuo Yamamori

**Affiliations:** 1 Division of Brain Biology, National Institute for Basic Biology, Okazaki, Japan; 2 Department of Molecular Genetics, Institute of Biomedical Sciences, Fukushima Medical University School of Medicine, Fukushima, Japan; 3 Department of Morphological Brain Science, Graduate School of Medicine, Kyoto University, Kyoto, Japan; 4 Department of Neurochemistry, Graduate School of Medicine, The University of Tokyo, Tokyo, Japan; 5 Research Center of Experimental Medicine, International University of Health and Welfare, Ohtawara City, Japan; 6 The Graduate University for Advanced Studies (Sokendai), Hayama, Japan; Institut Curie, France

## Abstract

We are interested in identifying and characterizing various projection neurons that constitute the neocortical circuit. For this purpose, we developed a novel lentiviral vector that carries the tetracycline transactivator (tTA) and the transgene under the TET Responsive Element promoter (TRE) on a single backbone. By pseudotyping such a vector with modified rabies G-protein, we were able to express palmitoylated-GFP (palGFP) or turboFP635 (RFP) in corticothalamic, corticocortical, and corticopontine neurons of mice. The high-level expression of the transgene achieved by the TET-Off system enabled us to observe characteristic elaboration of neuronal processes for each cell type. At higher magnification, we were able to observe fine structures such as boutons and spines as well. We also injected our retrograde TET-Off vector to the marmoset cortex and proved that it can be used to label the long-distance cortical connectivity of millimeter scale. In conclusion, our novel retrograde tracer provides an attractive option to investigate the morphologies of identified cortical projection neurons of various species.

## Introduction

The cerebral cortex of mammals consists of various types of projection neurons that are aligned in layers [1], [2]. These projection neurons exhibit characteristic morphology and intrinsic connectivity, which lay basis for the canonical lamina circuit common across areas and species [3]. Morphological studies of the cortical projection neurons date back to the invention of the Golgi silver impregnation technique, which enabled the first detailed analyses of axons and dendrites decorated with spines and boutons. The “Golgi-like” staining of targeted projection neurons was then made possible by dye-filling technique combined with retrograde tracers and/or electrophysiological recordings (e.g., [4], reviewed in [5]). Much of our current knowledge on the morphology and connectivity of cortical projection neurons was obtained by variations of such single-cell analysis. Recently, however, a new generation of viral-based retrograde tracers is becoming a useful option to analyze the morphology of a defined set of projection neurons.

Neurotropic viruses such as the rabies and pseudorabies viruses infect neurons at the terminals, transported back to the cell body, replicate, and spread across synapses for the next round of infection [6]–[8]. Using the retrograde infectivity of rabies virus, two types of monosynaptic retrograde tracer have been designed. One is the glycoprotein-deficient rabies vector carrying EGFP, which can retrogradely transduce and replicate within neurons to produce bright green fluorescence, but do not spread beyond the primary infection [9]. The other is the utilization of the glycoprotein of the rabies virus (RV-G) as the envelope of the lentiviral vectors (pseudotyping), which confers retrograde infectivity [10]–[13]. Because lentiviral vectors are designed not to replicate nor become infectious, they can also be used for stable monosynaptic labeling. Despite the advantage of the lentiviral vectors in terms of safety, the ease of handling and the low toxicity to the infected cells, its practical use as the retrograde vector has been limited due to low rate of retrograde infection. Recently, however, the situation changed dramatically by the invention of fusion envelope glycoproteins composed of parts of RV-G and vesicular stomatitis virus glycoprotein (VSV-G) [14]–[17]. The high rate of retrograde gene transfer by this new generation of vector system has many potential applications in the field of neuroscience. One caveat of this vector system is that transgene expression is not high enough to visualize fine morphological structures, such as boutons and spines, even when we used strong viral-derived promoters [12].

To overcome this limitation, we incorporated the “Tet-Off system”, which was previously shown to be very effective in amplifying the transgene expression from the lentiviral vector [18]. The Tet-Off system requires two components, namely, the tetracycline transactivator (tTA) under the cellular promoter and a transgene under the TET responsive element (TRE). We constructed a novel lentiviral vector that carries these two components within a single backbone by replacing the two transcription units of a SARE reporter [19]. Using this vector system to express membrane-targeting GFP (palGFP) and RFP (TurboFP635), we were able to visualize fine dendritic and axonal processes originated from selected subtypes of cortical projection neurons.

## Materials and Methods

### Ethics Statement

All the experiments were conducted in accordance with the guideline of the National Institutes of Health, and the Ministry of Education, Culture, Sports, Science and Technology (MEXT) of Japan, and were approved by the Institutional Animal Care and Use Committee of National Institutes of Natural Sciences. We made all efforts to minimize the number of animals used and their suffering.

### Plasmid construction

**Figure 1:**
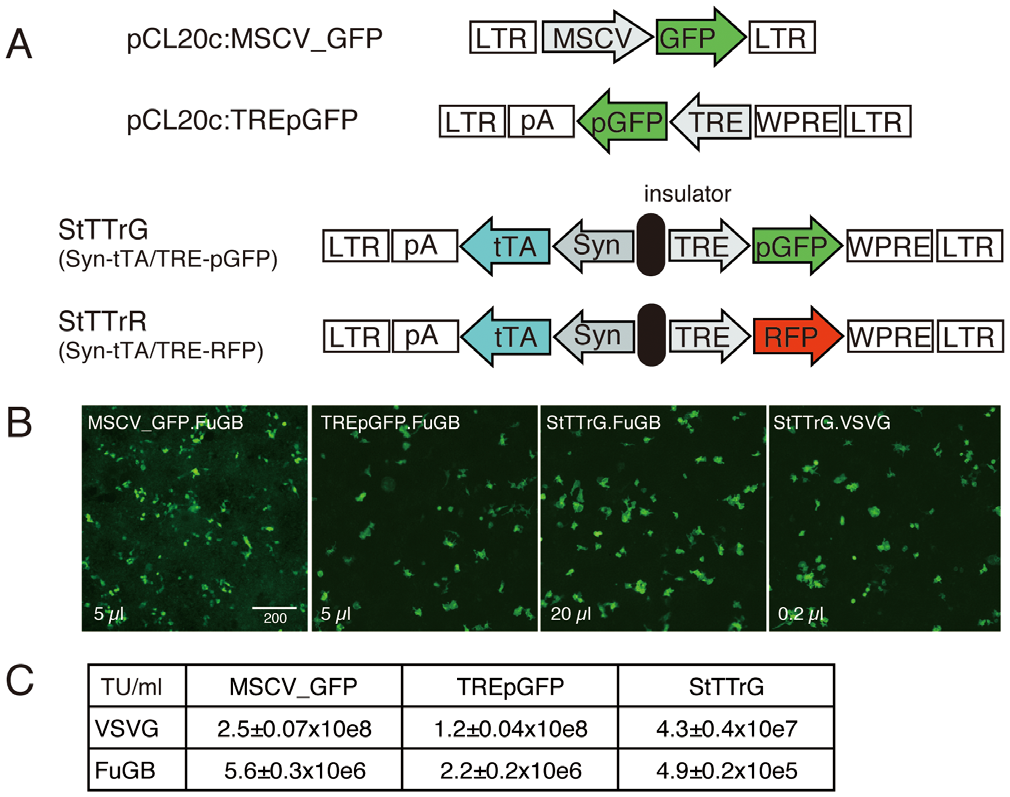
Double unit TET-Off lentiviral constructs for high-level transgene expression. (A) Schematic representation of the lentiviral vector constructs tested for packaging efficiency. MSCV_GFP and TREpGFP served as the control for the double unit vectors, StTTrG and StTTrR, which contain tTA under the human synapsin I promoter (Syn) as well as palmitoylated GFP (pGFP) or turboFP635 (RFP) under TRE promoter, separated by a chick HS4 insulator. (B) Examples of raw data for titration experiments. HEK293T cells seeded on a glass coverslips were exposed to the indicated amounts of the lentiviral supernatants for 24 hours, transfected with tTA-expressing plasmid (except MSCV_GFP) and imaged for cell counting. GFP signal was so strong that no antibody detection was required. For MSCV_GFP, we had to enhance the GFP fluorescence by antibody staining. Bar: 200 µm. (C) Infectious titers of the lentiviral vectors pseudotyped either with VSVG or FuG-B.

The constructs used in this study are schematically shown in Fig. 1. pCL20c:MSCV_GFP was previously reported [12]. pCL20c:TREpGFP was constructed by cloning the NheI-Asp718 fragment of TpGB [18] into the MluI-NotI locus of pCL20c:MSCV_GFP. StTTrG was constructed by a series of plasmid cloning, by which a portion of STB [18] was PCR-amplified to be cloned into the AscI-XbaI site of SARE-ArcMin lentiviral vector [19], replacing the SARE-d2EGFP with synapsinI-tTA, followed by cloning of MluI-ClaI fragment of TpGB (containing TRE-palGFP) into the BamHI-XhoI locus. StTTrR was constructed similarly except that the XhoI-SmaI fragment of TpGB was cloned into the XhoI-HpaI site at the last step, replacing the PGK promoter with TRE.

### Production of lentiviral vectors

Small scale preparation of the lentiviral vectors was performed by transient transfection of HEK293T cells with the mixture of the transfer, envelope, and packaging plasmids [12] using Lipofectamine 2000 (Invitrogen) as in [18]. The transfection media were replaced after 8 hours with DMEM containing 10% fetal bovine serum and 0.01 mM forskolin. The culture supernatant containing the viral particles was collected 48 hours post-transfection and was used without concentration for the titration experiment.

The infectious titers of the lentiviral preparations were determined by infecting HEK293T cells with various dilutions. To seed HEK293T cells, round coverslips (Matsunami, 13 mm, No. 2) were lysine-coated by soaking in 5 µg/ml solution of poly-D-lysine (0.1 mg/ml, Sigma, P-0899) in 60 mm culture dish. HEK293T cells were seeded onto this lysine-coated dish at a density of 1.24×10e6 cells/5 ml of medium. After 24 hours, each coverslip was transferred to a 24-well plate with 300 µl of the medium. The diluted viral preparations were then added to the medium and incubated for 12 hours, followed by transfection by a plasmid carrying tTA under the CMV promoter, using Lipofectamine 2000. After 8 hours of transfection, the medium was changed to DMEM containing 10% fetal bovine serum and incubated for further 24 hours. Then the cells were fixed by 4% paraformaldehyde and washed by PBS. The cells infected with the lentiviral vectors carrying the TRE-palGFP or TRE-RFP unit exhibited bright fluorescence in the presence of exogenous tTA (Fig. 1B). To measure the titer of MSCV_GFP vector, transfection was not performed and the incubation was continued for 60 hours after the viral medium was removed, during which the medium was changed once. For this vector, GFP expression was enhanced by immunofluorescence staining for counting. The number of the positive cells was determined semi-automatically using Image-Pro Plus image analysis software (Media Cybernetics). In our protocol, HEK293T cells proliferate after infection, which increase the number of the positive cells. On the other hand, the divided cells remain touched with each other and were often regarded as a single cell in the automatic cell counting. For these reasons, the infectious titer should be considered as a measure of relative infectivity, although we obtained reproducible results as long as we stick to these conditions.

The lentiviral preparations used for in vivo injection experiments were produced in large scale and purified by ion exchange column chromatography as previously described [16]. The titers of the lentiviral preparations were measured by RT-PCR as in the previous studies [14], [15]. They were in the range of 2.3–5×10e12 copies/ml.

### Viral Injection

Seven adult BL6 mice (12–20 weeks) were anesthetized with an intraperitoneal injection of ketamine/xylazine mixture (100 mg/kg, 10 mg/kg, respectively). A small hole was made in the skull using a dental drill. The virus was delivered by pressure injection using a glass micropipette (tip size of roughly 40 µm for cortical injection and 50–70 µm for subcortical injection) attached to a nanoliter 2000 injector connected to Micro4 controller (World Precision instrument). To inject virus into the brain, the dura was punctured using a tip of 27G needle, through which the glass pipette was slowly lowered to the target depth. Approximately 0.5 µl (for cortical injections) or 0.5–1 µl (for subcortical injections) of the viral solutions were injected at the rate of 0.1–0.2 µl/min. For co-injection experiment with the fluorescent tracer, the viral solution was mixed with one-fifth volume of Cholera Toxin Subunit B-Alexa Fluor 488 conjugate (CTB-Alexa488; final 0.2 mg/ml, Invitrogen, #C-22841) The pipette was held in place for 2 minutes before and after the injection. After retracting the glass micropipette, the hole was filled with Spongel, an absorbable gelatin sponge (Astellas Pharma Inc.) and the head skin was sutured. The mice were sacrificed three to four weeks after injection except in one case of one week waiting.

One New World marmoset monkey (*Callithrix jacchus*; three year old, male, weighing approximately 350 g) was used in this study. Following an intramuscular injection of ketamine/xylazine mixture (30 mg/kg, 1.3 mg/kg, respectively), sodium thiopental (25 mg/kg) was intraperitoneally injected to induce anesthesia, which was maintained by additional shot of ketamine (15 mg/kg), when needed. Heart rate and the rectal temperature were continuously monitored. The virus injection was performed in a similar manner as described above for mice. In this marmoset, StTTrR/FuG-B was injected into four sites in the left hemisphere (AP+2.25 mm, left 3 mm; AP+2 mm, left 5 mm; AP-7.5 mm, left 2 mm; AP-7.5 mm, left 4 mm; all at depth 0.7 mm). In addition, StTTrG/FuG-B was injected to two sites in the left hemisphere (AP+2 mm, left 4 mm; AP-7.5 mm, left 3 mm) and CTB-Alexa488 and CTB-Alexa555 were injected into several sites in the right hemisphere for the purpose of other studies. The current study concerns only the result of transduction by StTTrR. The marmoset was sacrificed three weeks after injection by transcardial perfusion of 0.9% NaCl, followed by 4% paraformaldehyde/0.1 M phosphate buffer (pH7.4).

### Immunostaining

Mice and the marmoset were anesthetized with sodium pentobarbital and perfused transcardially with 0.9% NaCl, followed by fixation with 4% paraformaldehyde in 0.1 M phosphate buffer, pH 7.4. For immunoperoxidase detection, the sections were incubated with 1% H2O2 in TBS for 30 min to suppress the intrinsic peroxidase activity and blocked with 10% fetal bovine serum, 2% bovine serum albumin, and 0.5% Triton ×100 in TBS, pH7.4, followed by overnight incubation with the primary antibody for GFP (1∶20000; [20]) or for turboFP635 (1∶5,000; rabbit polyclonal, Evrogen, #AB233) at 4°C. After incubation with the biotinylated secondary antibody (anti-rabbit IgG, 1∶2000; Jackson ImmunoResearch), the immunoreactive signals were visualized by use of a Vectastain Elite ABC kit (Vector Laboratories) with 3,3-diaminobenzidine (DAB) and nickel as chromogens.

For double immunofluorescence, the sections (20–40 µm thick) were incubated with a primary antibody for turboFP635 and for NeuN (1∶2,000; mouse monoclonal, Millipore #MAB377) at 4°C overnight, followed by secondary antibody detection (Cy3 conjugated anti-rabbit IgG 1∶2000; Jackson ImmunoResearch, #711-165-152, and Alexa Fluor 488 conjugated anti mouse IgG 1∶2000; Invitrogen, A11029), and counterstained with Hoechst 33342 (1∶1000; Molecular Probes). The fluorescent images were captured by Olympus DP71 digital camera attached to BX51 microscope (Olympus). The confocal images were taken by Nikon confocal laser microscope system A1. Maximum intensity projection images for the confocal data were created by NIS-Elements imaging software (Nikon). All the images were processed by Adobe photoshop for proper contrast for presentation.

## Results

### Double unit Tet-Off vector can induce high-level expression in the mouse cortex

The effectiveness of the double unit strategy for transduction of tTA and TRE-GFP has been shown in the previous study [18]. The lentiviral constructs in this study, however, exhibited very low infectious titers to be used for RV-G pseudotyping. Because the double unit vector harboring the SARE-GFP and PGK-RFP units separated by a cHS4 insulator sequence [19] exhibited a relatively high titer (data not shown), we tested whether it can be adapted for use with the Tet-Off system. We constructed two vectors for expression of GFP and RFP (Fig. 1A; StTTrG and StTTrR). Both vectors contain tTA under human synapsin I promoter, which drives neuronal expression [18], [21]. Separated by a chicken cHS4 insulator sequence, GFP (for StTTrG) or RFP (for StTTrR) under TRE promoter was placed in opposite direction as the second transcription unit. In case of StTTrG, GFP was tagged with palmitoylation signal for better visualization of membranous structure [20].

To examine the efficacy of this double unit vector system, we produced the lentiviral vectors pseudotyped with either standard VSVG or fusion glycoprotein B type (FuG-B) composed of the extracellular and transmembrane domains of RV-G and the cytoplasmic domain of VSV-G [15], and measured their infectious titer to HEK293T cells. Since synapsin I promoter did not work well in this cell line, we supplied tTA by plasmid transfection and counted the number of the GFP-positive cells as the infected cell (Fig. 1B, see materials and methods for detail). For comparison, we used two more constructs for lentiviral production (Fig. 1A). pCL20c:MSCV_GFP was used as the control for a standard single unit vector [12]. pCL20c:TREpGFP was constructed by changing the lentiviral backbone of TpGB [18] to that of pCL20c:MSCV_GFP. As shown in Fig. 1c, both these control constructs yielded high infectious titers without further concentration, when pseudotyped with VSVG. In comparison, StTTrG exhibited 5.8 and 2.8-fold decrease in titer compared with these constructs (Fig. 1C). When pseudotyped with FuG-B, the titers for the three vectors, MSCV-GFP, TREpGFP and StTTrG, decreased by 45, 55, and 88-fold, respectively. Although this is a large decrease, we expected that we can still get in vivo infection by concentrating the viral particles.

**Figure 2:**
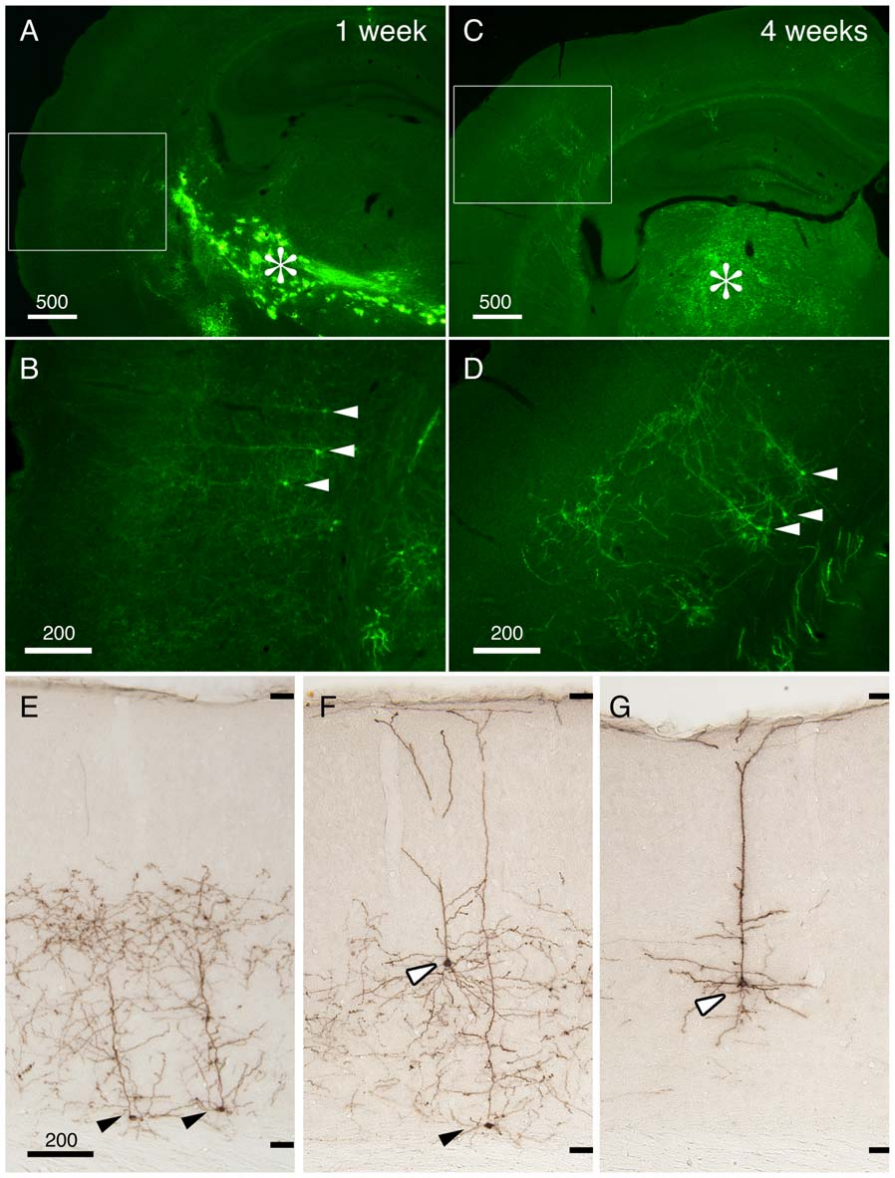
Retrograde infection of the corticothalamic cells by StTTrG/FuG-B vector. StTTrG/FuG-B vector was injected into the mouse thalamus. (A–D) After one week (for panels A and B) or four weeks (for panles C–G), the mouse was perfusion fixed and sliced at 40 µm thickness. The boxed areas in panels A and C were magnified in panels B and D, to show infection of corticothalamic neurons in layer 6 (indicated by arrowheads). The injection site is indicated by the asterisk (*). These images show the original green fluorescence of the infected cells. (E–G) The immunoperoxidase detection of the GFP-expressing cells. Neurons in layer 6 (filled arrowheads) and layer 5 (open arrowheads) extended dense processes in the deep layers but not in the upper layers. The upper and lower limits of the cortical grey matter are shown on the right side. Bar: 500 µm for panels A and C, 200 µm for others.

In Fig. 2, we concentrated and purified StTTrG/FuG-B vector to be injected into the thalamus. After one week of viral injection, we were able to observe the labeling of cortical neurons in the remote site, which is considered to be the corticothalamic cells projecting to the site of viral injection (Fig. 2B, arrowheads). At this time point, the GFP fluorescence was still weak, but the fine processes were already visible without immunological enhancement of the signal. In addition to such retrograde labeling, we observed strong GFP fluorescence at the site of injection and along the fiber pathways (Fig. 2A, asterisk). The infected cells in these regions did not look like neurons, based on lack of long processes and the shape of the cell body (data not shown). Four weeks after the injection, the GFP fluorescence became strong and the fine neurites were now clearly observed to extend from the layer 6 neurons (Fig. 2D, arrowheads). On the other hand, the staining at the thalamic injection site became much less conspicuous (Fig. 2C, asterisk). The cells that showed strong GFP fluorescence at the injection site at one weak could be reactive astrocytes, which transiently exhibited leak expression of the synapsin promoter.

To examine the morphology of the retrogradely labeled cortical cells in more details, we enhanced GFP signal by anti-GFP antibody. Fig. 2E–G demonstrates three types of corticothalamic neurons that were visualized by our StTTrG/FuG-B vector. The most common type was the short pyramidal cells of layer 6 that extend neurites only to the middle of cortical layers (Fig. 2E, black arrowhead). We also observed tall pyramidal cells of layer 6 (Fig. 2F, black arrowhead) and 5 (Fig. 2F and G, white arrowhead) that extend the apical dendrites to reach layer 1. The overall appearance of the neurite arborization of this group of cells is consistent with those reported previously [22]–[26]. Thus, StTTrG/FuG-B vector can be used *in vivo* for visualization of the cortical projection neurons.

### Layer specific distribution of the corticothalamic neurites

In the viral method, many cells are infected simultaneously by a single injection. For this reason, it is usually difficult to isolate the neurites of a single cell from other infected cells. Instead, the viral method gives us a population data for a defined group of cells. In the following sections, we illustrate the usefulness of such technique taking examples from the labeling of the corticothalamic, corticocortical and corticopontine cells.

**Figure 3:**
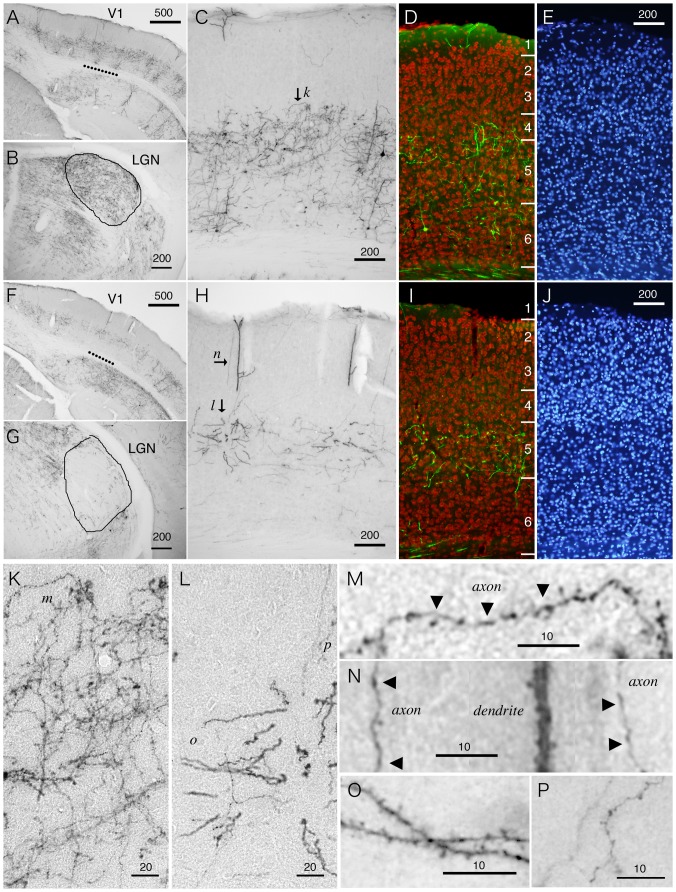
Fine morphology of the corticothalamic neurites of V1 revealed by the viral vector. StTTrG/FuG-B vector was injected into the thalamus and the retrogradely labeled cells were examined in V1 by immunoperoxidase or fluorescence detection. (A–E) Retrograde labeling of the corticothalamic cells with an injection involving the LGN. The break line in panel A indicates V1, which is magnified in panel C. As shown in panel B, we observed dense terminals in the LGN in this injection. (D and E) The section was counterstained with NeuN antibody (red) and Hoeschst nuclear staining (blue) to determine the exact lamina position for dendritic and axonal elaboration. (F–J) Retrograde labeling of the corticothalamic cells with an injection outside the LGN. In panel F, we observed the neurite staining in layer 5 that covers a large region. The break line in panel F indicates V1, which is magnified in panel H. Note the lack of labeling in layer 6 cells only within V1, which is considered to correspond to the lack of dense terminals in the LGN in panel G. (I and J) Same as panels D and E. (K–P) Magnified views of the corticothalamic processes shown in panels C and H. The lower case letters in each panel indicates the positions of the panels that enlarged the region of interest. Bar: 500 µm for A and F; 200 µm for B–E, G–J; 20 µm for K and L; 10 µm for M–P.

In Fig. 3, we compared two cases of thalamic injections of StTTrG/FuG-B vector. In the first case, the injection involved a large thalamic area including the lateral geniculate nucleus (LGN), which is manifested by its dense innervation in this thalamic subnucleus (Fig. 3B). As we mentioned earlier, there exist three types of corticothalamic cells with cell bodies located in layers 5 and 6 (Fig. 2E–G). In V1 of the LGN-injected sample, we were not able to distinguish these cell types, because the cells were dense and the dendrites were oblique to the sectioning (Fig. 3C). Nevertheless, the layer-specific distribution of the cell bodies and plexuses of neurites were quite conspicuous. It was especially notable that there is a ceiling of neurite extention in the middle of cortical layers. The counterstaining with NeuN antibody and Hoechst 33342 nuclear dye indicated that the majority of the neurites do not cross the border between layers 4 and 5, although some neurites do elaborate in layer 4 (Fig. 3D and E). In contrast to this first case, the second case of injection avoided the LGN (Fig. 3G). The surrounding thalamic subnuclei, such as LD, contained the terminals. In V1 of this “non-LGN” case, we could not find layer 6 cells. Only layer 5 cells with apical dendrites reaching layer 1 were labeled (Fig. 3F and H). The neurites from this group of cells were observed confined to layer 5, except those that run vertically to reach layer 1 (Fig. 3H–J). The layer 5 cells in this case are considered to correspond to the “drivers” proposed by Sherman and Guillery [27].

Next, we examined whether we can observe fine structures such as boutons and spines, which will aid in identification of axons and dendrites. In Fig. 3K and L, we magnified the layer 5 plexus of the LGN and non-LGN injected samples. In Fig. 3K, the plexus in layer 5 (Fig. 3C, arrow k) contained many thin and beaded neurites, which are considered to be the axon collaterals with boutons (Fig. 3K and M). In contrast, the processes in layer 5 of Fig. 3L were thick and decorated with spines (Fig. 3L and O). These are most likely the basal dendrites coming from the layer 5 cell in the adjacent section. The thin and beaded neurites, the putative axon collaterals, were rather scarce (Fig. 3P). The thick apical dendrites that reach layer 1 were typically decorated with spines (magnified in Fig. 3N). There were also axons with boutons that run along this dendrite to reach layer 1 (Fig. 3N).

In conclusion, LGN and non-LGN injection of the TET-Off vector resulted in bulk labeling of the corticothalamic cells, which revealed high layer-specificity of neurite extensions for this group of cells. We also showed that StTTrG/FuG-B vector can reveal the fine morphology of neuronal processes.

### Co-injection of StTTrR/FuG-B vector and fluorescent tracer into M1 for comparison of cortical labeling

In the thalamic injection experiments, the number of the labeled cell bodies was not very high, despite extensive labeling of the neuronal processes. To determine the efficiency of labeling, we next injected the TET-Off vector together with the classic fluorescent tracer. In this experiment, we used StTTrR/FuG-B lentiviral vector, which expressed RFP with no tag. We considered that this version of the TET-Off vector can visualize the cell bodies better, because the palmitoylated GFP expressed from StTTrG accumulates in the surface membrane.

**Figure 4:**
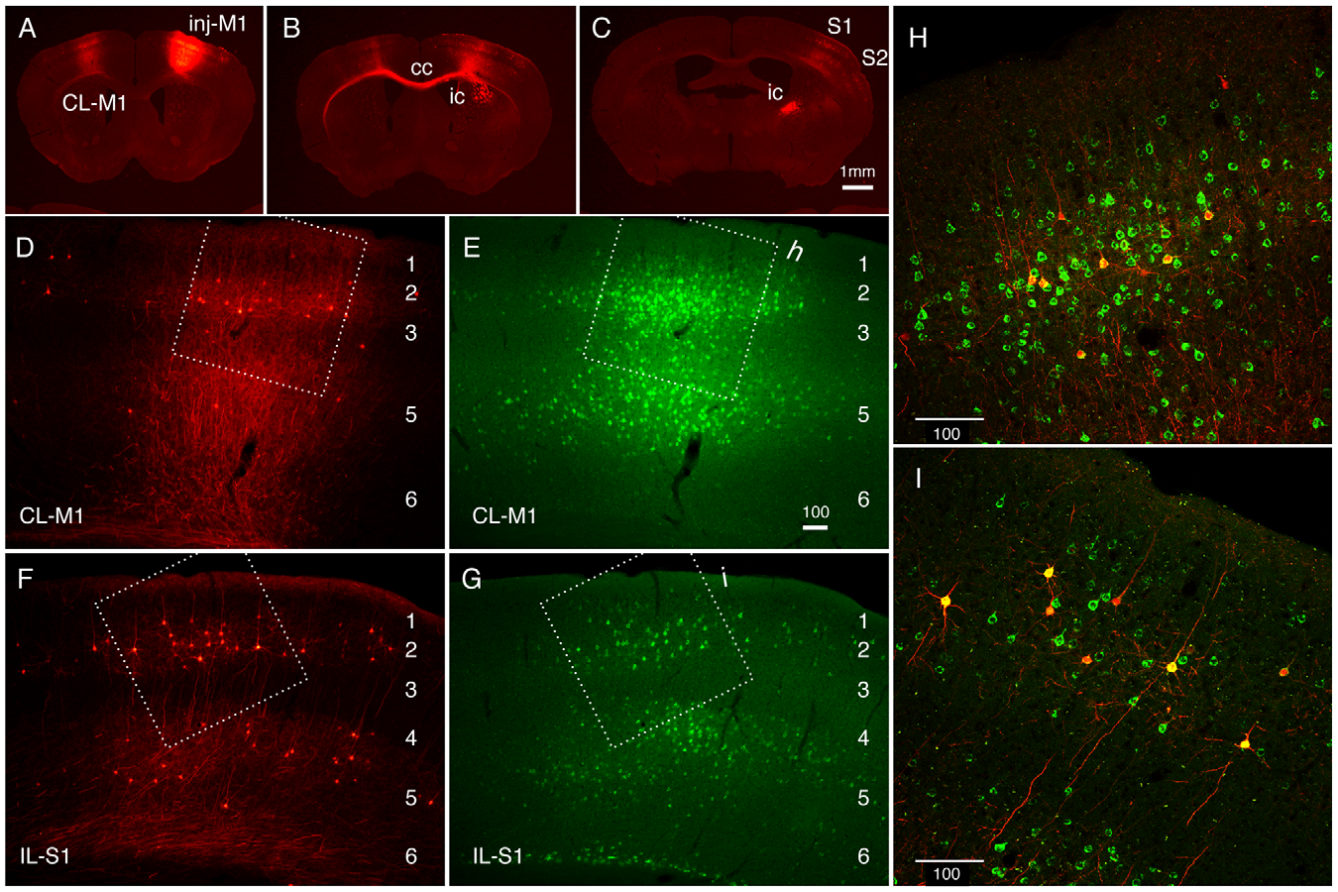
Retrograde infection of the corticocortical cells by StTTrR/FuG-B vector in comparison with CTB-Alexa488. StTTrG/FuG-B vector was mixed with CTB-Alexa488 and injected into mouse M1. (A–C) Low magnified images of the RFP fluorescence. The injection site (inj-M1) exhibited bright fluorescence due to local infection. The axons originating from this site are labeled in the contralateral M1 (CL-M1), corpus callosum (cc), internal capsule (ic), S1 and S2. The dot-like labeling in CL-M1, S1 and S2 are retrogradely infected cells. (D and E) Magnified views of the contralateral M1. The cells retrogradely labeled by RFP (D) and CTB-Alexa488 (E) are shown. The boxed region is magnified in panel H. (F and G) Magnified views of the ipsilateral S1. The boxed region is magnified in panel I. (H and I) Confocal images of the regions shown in panels D–G. The merged images of RFP (red) and CTB-Alexa488 (green) are shown. RFP signals shown in this figure is not enhanced by antibody detection. Bar: 1 mm for A–C; 100 µm for D–I.

In Fig. 4, we show the result of co-injection of StTTrR/FuG-B vector and CTB-Alexa488 into mouse M1. In this injection, the injection center was clearly identified by heavy loading of both CTB-Alexa488 and RFP, which were restricted within the cortical layers (Fig. 4A). As expected, CTB-Alexa488 retrogradely labeled the neurons dispersed in various regions, including contralateral M1, ipsi- and contralateral S1 and S2, claustrum, and thalamus, consistent with the previous reports [28]–[30]. Although the efficiency was low, the StTTrR-infected neurons were observed in all of these brain regions (data not shown). Nevertheless, the efficiency of retrograde labeling was variable. For example, the layer 3 neurons in the ipsilateral S1 exhibited relatively high ratio of the RFP-positive neurons among the CTB-positive cells (approximately 20%), compared with that in the contralateral M1 (approximately 5%; Fig. 4D–I). Furthermore, the low efficiency of the layer 5 labeling was apparent just by visual inspection, although a few cells were still labeled by RFP (compare Fig. 4D and E or Fig. 4F and G). From these observations, we conclude that (1) the infection efficiency of the retrograde viral vector is low and variable (around 5–20%) and (2) despite low efficiency, the retrograde infection pattern is consistent with that of the fluorescent tracer.

### Characterization of the retrograde labeling by StTTrR vector

**Figure 5:**
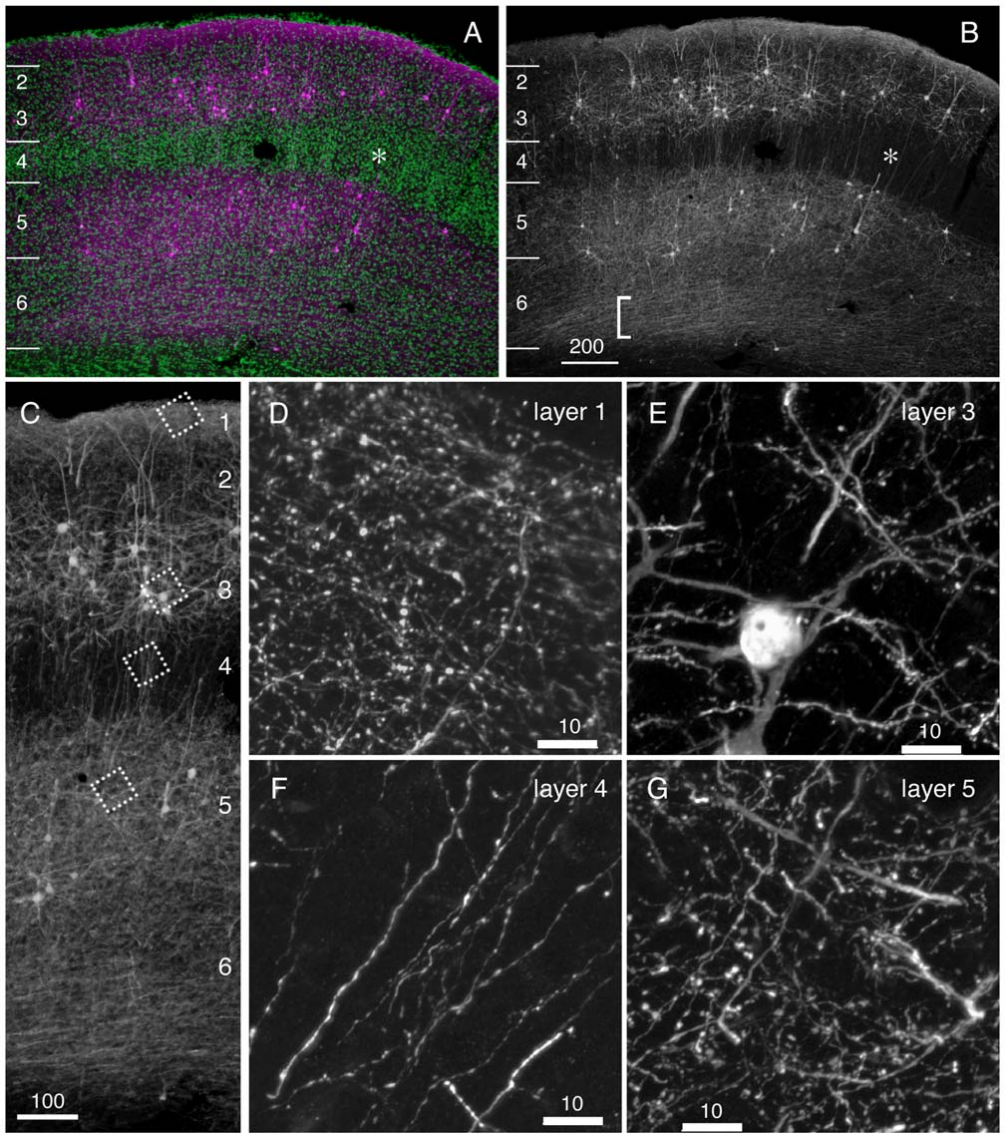
Labeling of the ipsilateral cortical projection neurons by StTTrR/FuG-B vector. Fine morphology of dendritic and axonal processes for the corticocortical neurons shown in Fig. 4 was imaged by confocal microscopy. (A) A low power view of S1 ipsilateral to the injection site. To increase the sensitivity of detection, RFP signals were enhanced by immunostaining of turboFP635 (magenta). To determine the exact layer of neurite elaboration, the sections were counterstained with Hoechst nuclear staining (green). Asterisks indicate layer 4 of the barrel field. (B) Same as A. Only the RFP signals are shown. Note the absence of RFP signals in layer 4. The fibers at the bottom of layer 6 may be forward projection fibers (bracketed), which originated from the injection site. (C) A higher power view of panel B. The boxed regions in layers 1, 3, 4 and 5 are magnified in panels D–G, respectively. Bar: 200 µm for panels A and B; 100 µm for panel C; 10 µm for panels D–G.

**Figure 6:**
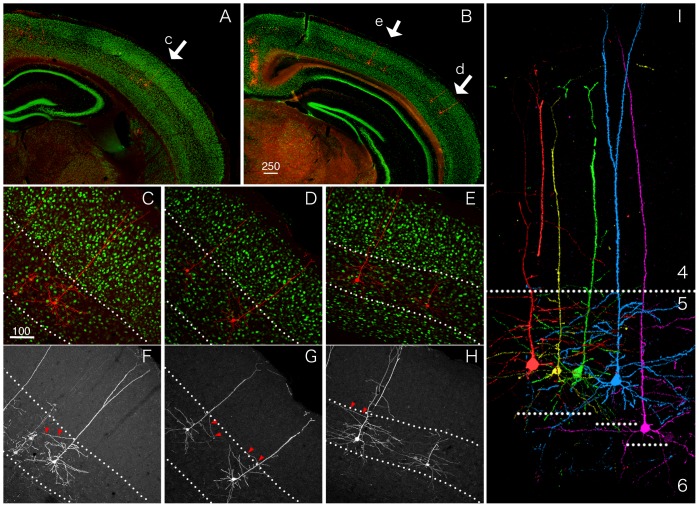
Retrograde labeling of the corticopontine cells revealed layer 5-restricted elaboration of the dendritic processes. StTTrR/FuG-C vector was injected into the pontine nuclei. (A and B) Low power views of the cortical sections stained for RFP (red) and NeuN (green). In this injection, retrogradely labeled corticopontine cells were dispersed over wide cortical areas. The areas denoted by arrows are magnified in panels C–E to show the morphology of these neurons. (C–E) Higher power views of the corticopontine cells shown in panels A and B. The break lines indicate the borders above and below layer 5, which can be accurately determined based on the NeuN staining. (F–H) Same as C–H. Only the RFP signals are shown. Note that the horizontal branching from the apical dendritic shafts are mostly contained within layer 5. (I) The images of the corticopontine cells extracted from five different regions. These images are aligned to the position of the layer 4/5 border with no size adjustment. The break lines below the cell bodies indicate the layer 5/6 borders for each cell. Bar: 250 µm for A and B; 100 µm for C–H.

Despite lack of subcellular targeting signals, RFP expressed at high level from StTTrR vector visualized the morphology of the infected neurons. Below, we take examples from the cortical (Fig. 4 and 5) and pontine injections (Fig. 6) to explain how the corticofugal cells were labeled by this TET-Off vector.

In the case of cortical injection, the heaviest labeling of RFP occurred at the injection site (Fig. 4A). Due to this local labeling, the forward projections, such as those that go through corpus callossum (Fig. 4B, cc) and internal capsule (Fig. 4B and C, ic) were strongly labeled by RFP. In addition, numerous branches were observed to emanate from the injected site at higher magnification. Therefore, the neurites observed at the areas of retrograde infection contained such forward projections. The area shown in Fig. 5 corresponds to S1, which has reciprocal connections with the ipsilateral M1 [28]. In this area, we observed especially dense neurite networks in layers 1, 3, 5 and 6 (Fig. 5B). Among these, the fibers present in the lower part of layer 6 (Fig. 5B, bracketed) and the thin processes in layer 1 that have many boutons (Fig. 5D) may be forward axonal projections from the ipsilateral M1, considering a previous report of AAV injection into M1 [28]. On the other hand, the relatively thick processes that populate layer 3 were decorated with spines (Fig. 5E) and should be the dendrites of the M1-projecting neurons (Fig. 5C and E). The dense plexus in layer 5 consisted of both axons and dendrites (Fig. 5G). We consider that the axons in this layer are a mixture of M1 and local origin. Compared with these layers, we observed relatively sparse neurites with no spines or boutons in layer 4 (Fig. 5B and F). Thus, although the reciprocal connections between cortical areas confounded the interpretation of the results of retrograde labeling, the layer-specific nature of the corticocortical connectivity was visualized by our vector system.

As another example of cortical projection neurons, we selected the corticopontine neurons for analysis. In the experiment shown in Fig. 6, StTTrR lentiviral vector was enveloped in FuG-C, another VSVG/RVG fusion envelope [14]. In our preliminary experiments, FuG-C envelope showed comparable or slightly more efficient retrograde labeling compared with the FuG-B envelope in the thalamic injection (data not shown). As expected, we observed strong expression of RFP in the cell body and dendrites of the corticopontine neurons dispersed over the cortical areas, four weeks after the pontine injection. Consistent with the previous reports [31], the corticoponine cells positioned in the lower half of layer 5. Furthermore, we noticed that elaboration of the horizontal dendritic branches from the main apical dendritic shaft is layer-specific (Fig. 6 F–H). This point is highlighted in Fig. 6I, in which five corticopontine cells were aligned at the layer 4–5 border. In this figure, it is obvious that the horizontal dendritic branches are conspicuous within layer 5 but not in the upper layers. In particular, note the presence of long horizontal branches just around the border between layers 4 and 5 in these cells (Fig. 6F–H; red arrowhead). These branches extend as if they avoided entering layer 4. Such long branches were rarely observed above layer 4. Because the images shown in Fig. 6 were obtained from the section of only 40 µm thickness, lack of long branches do not warrant the complete absence of such branches. Nevertheless, we can safely conclude that the frequency of apical dendritic branching diminishes greatly at the layer 4/5 border.

### Retrograde Tet-Off vector can be used in the marmoset brain

**Figure 7:**
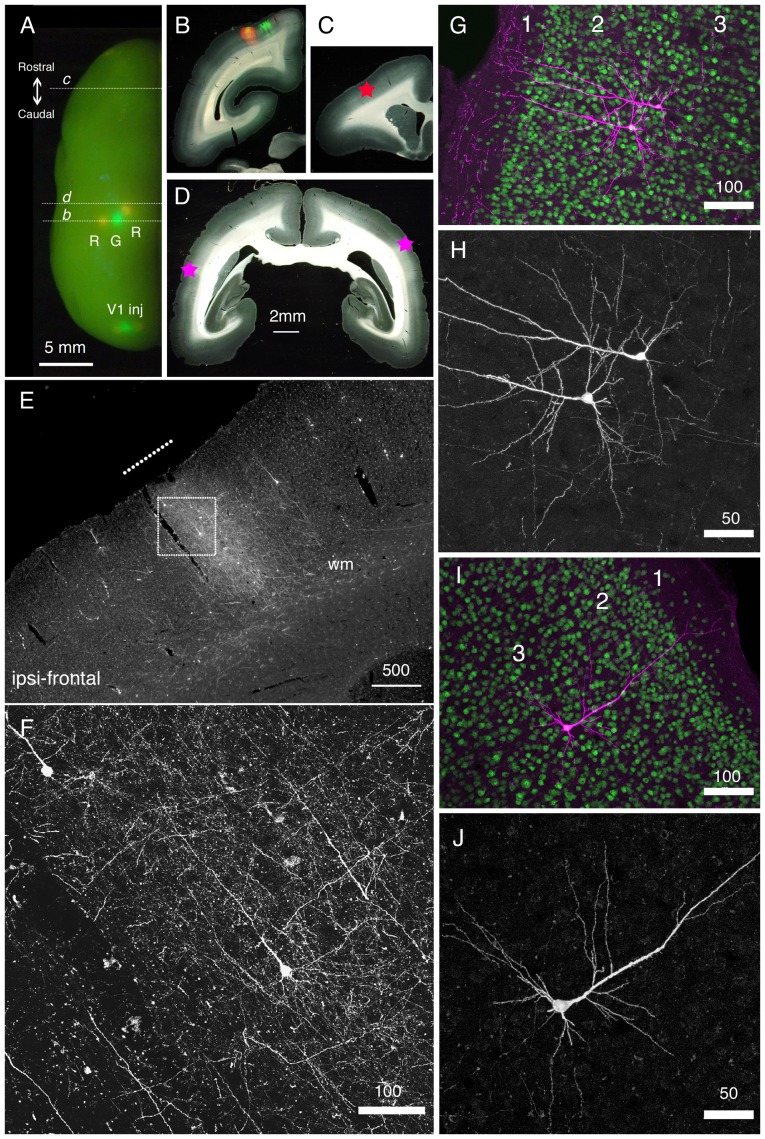
Long-distance cortical connection of the marmoset brain visualized by StTTrR/FuG-B vector. (A) The left hemisphere of the marmoset brain with cortical vector injections is shown from above. StTTrR/FuG-B vector was injected into two sites in the parietal areas and two sites in V1 and StTTrG/FuG-B vector was injected into one site in the parietal area and one site in V1, as indicated. The injection sites were visualized by LED illumination. (B–D) The coronal sections at the positions indicated in panel A. The fluorescent image is overlaid on the dark field image of the section in panel B. The asterisk in panel C shows the position of the image shown in panel E. The asterisk in the left side of the section in panel D corresponds to the ipsilateral side. (E) The frontal cortex ipsilateral to the vector injection. At this plane, the forward projection fibers of RFP were visible (shown by a white line in panel E), together with retrogradely labeled cell bodies. (F) Confocal image of the boxed region in panel E. Dense axonal fibers and dendrites were visible. This is the fluorescence image of RFP with no antibody enhancement. (G–J) Confocal images of the corticocortical neurons. RFP signals are enhanced by immunofluorescence (magenta) and counterstained with NeuN antibody (green) in panels G and I. Only the RFP signals are shown in panels H and J. The two neurons in panels G and H and one neuron in panels I and J are ipsilateral and contralateral to the injection site, respectively. Bar: 5 mm for A; 2 mm for B–D; 500 µm for E; 100 µm for F, G and I. 50 µm for H and J.

**Figure 8:**
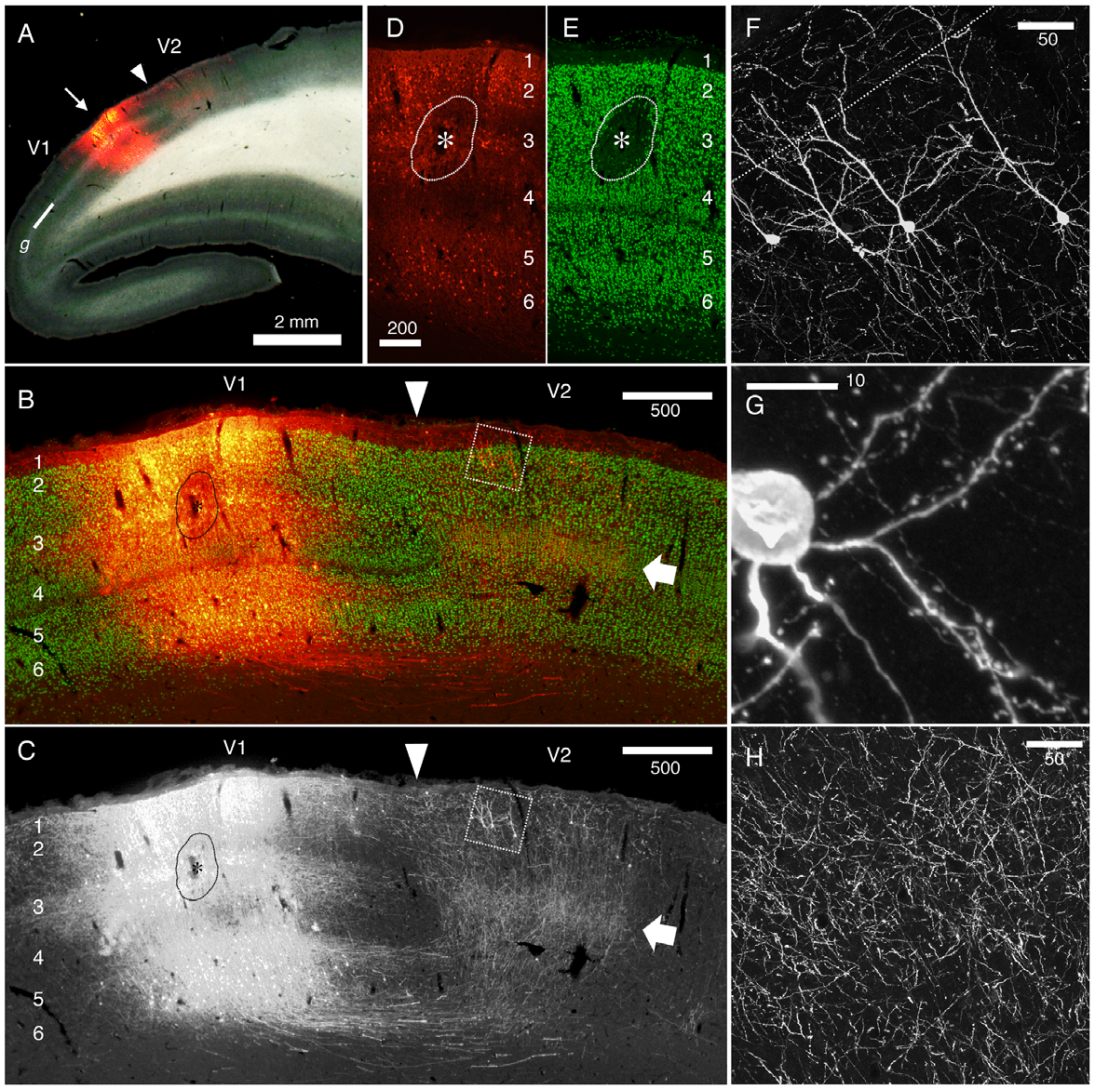
Reciprocal connectivity of marmoset V1 and V2 visualized by StTTrR/FuG-B vector. The caudal block of the marmoset brain shown in Fig. 7 was parasagittally sectioned to visualize the reciprocal connectivity between V1 and V2. (A) Dark field image overlaid with RFP fluorescence indicates the injection site. The V1/V2 border (shown by the arrowhead) was clearly delineated by the presence of the striate of Gennari (g). The arrow indicates the injection site. (B and C) The RFP signals around the injection site (red) are shown with NeuN conterstaining (green). The arrow indicates the plexus of V1 terminals in layer 4. The boxed region contains the retrogradely labeled V2 neurons with feedback projection to V1, which is magnified in panel F. The asterisk indicates the core of injection, where NeuN expression is lost by local damage. Only the RFP signals are shown in panel C. (D and E) RFP signals (red) and NeuN stains (green) in the injection center. The contrast for the RFP signals is adjusted so that the cell bodies of the infected neurons can be delineated. Note that the lamina positions of the infected cells are restricted mostly to layers 2, 3 and 6. (F–H) Confocal images of RFP in V2. RFP signals are enhanced by immunofluorescence. Panel F shows the cell bodies and dendrites of V2 neurons that project back to V1. Panel G shows the spines of the basal dendrites originating from one of these cells. Panel H shows the plexus of axon terminals concentrated in layer 4 of V2 (shown by the arrow). Bar: 2 mm for A; 500 µm for B and C; 200 µm for D and E; 50 µm for F and H; 10 µm for G.

So far, we have examined the utility of our retrograde Tet-Off vector in mice. We next injected our vector to the marmoset brain to examine if it can be used for the study of non-human primates. In Fig. 7A, we showed one example of marmoset injection, in which retrograde TET-Off vectors were injected to several cortical regions. In this figure, the left hemisphere of the vector-injected marmoset brain is displayed for localization of the injection sites. The strong fluorescence derived from StTTrR (denoted as R) and StTTrG (denoted as G) at the injection sites were observable by LED illumination from the surface. The vectors were injected into three neighboring regions in the parietal area (Fig. 7A and B) and three regions in V1 (Fig. 7A, V1inj; Fig. 8). By examining the coronally cut serial sections, we observed both green and red fluorescence derived from the TET-Off vectors in the remote sites. In this article, we show only the result of StTTrR. In Fig. 7E, we show a clear example of retrograde labeling in a part of the frontal cortex (star in Fig. 7C), which is 10 mm apart from the injection sites. We could follow the longitudinal fibers from the injection sites to this frontal area, where they turned up to exhibit a column-to-column connectivity (shown by a break line in Fig. 7E). Among the dense network of fibers, we detected several cell bodies that were retrogradely infected by StTTrR (Fig. 7F). Fig. 7F shows a confocal image with no antibody enhancement. Despite the dot-like autofluorescence, RFP signal was strong enough to be imaged without immunological enhancement. In addition to this frontal labeling, we could also detect the retrogradely labeled cells in the lateral regions of both hemispheres (Fig. 7D, star). Fig. 7G–J show the antibody-enhanced RFP counterstained with NeuN. These are layer 3 neurons projecting ipsilaterally (Fig. 7G and H) or contralaterally (Fig. 7I and J) to the injection site. As these examples show, our TET-Off vectors can be used to label the neurons separated by millimeter-scale axonal tracts.

As another example, we show the result of V1 injection of StTTrR/FuG-B vector in Fig. 8. V1 or striate cortex of marmoset can be delineated by the presence of the striate of Gennari (denoted as g) as in Fig. 8A. In this parasagittal section, the antibody-enhanced RFP counterstained with NeuN clearly demonstrated a dense network of V1–V2 innervations. The majority of this staining originated from the local infection within V1. In the center of infection, we observed loss of NeuN staining (Fig. 8D and E, bracketed by a break line and denoted as *), which may have been caused by physical damage of the pipet penetration, inflammation to lentiviral particles and/or too much transgene expression. Nevertheless, the RFP positive cells around this center did not show reduction of NeuN staining. These positive cells were predominantly found in layers 2, 3 and 6 (Fig. 8D and E), bypassing layers 4 and 5, suggesting that the infection is not random. It is possible that they are the retrogradely infected cells via the terminals located in the injection center in layer 3. Because of such V1 labeling, the forward projection fibers fromV1 to V2 were heavily stained. Consistent with the previous report [32], the projection to V2 terminated mostly in layer 4 (Fig. 8B and C; white arrows), where we observed thin fibers with bouton-like varicosities (Fig. 8H).

As expected from the reciprocal connectivity between V1 and V2, the retrogradely infected cells in V2 were found overlapped with the V1-to-V2 innervations. As was first noted by Rockland and Pandya [32], the “feedback” connections from V2 to V1 originated from both superficial and infragranular layers. In our injection experiment, we observed many RFP positive cells in layer 2 (Fig. 8B, rectangle), but much fewer cells in layer 6. Although the V1-fibers were present all across layers, the dendritic morphology of the layer 2 “feedback neurons” were visible as shown in Fig. 8F. At higher magnification, we could observe spines to decorate the dendrites of these neurons (Fig. 8G). In Fig. 8G, thin fibers with apparent bouton-like structure were also visible. They are considered to be either the axon terminals from V1 or the axon collaterals of the V2 neurons.

In summary, we proved that our retrograde TET-Off vectors can be transmitted through a long distance to label marmoset cortical projection neurons. Although the reciprocal connectivity made the axonal analysis complicated, the dendritic structures of the retrogradely infected cells were delineated at high magnification.

## Discussion

In this study, we produced and characterized a novel lentiviral-based retrograde tracer. The key of this vector system was the incorporation of the tTA gene and TRE-transgene components in a single vector backbone. High-level expression of the fluorescent proteins, thus achieved, enabled us to image the entire morphology of the retrogradely infected cell. Utilizing this system, we were able to visualize the layer-specific elaboration of neuronal processes for the identified cortical projection neuron subtypes of mouse and marmoset. To date, the standard method for morphological analyses of the cortical neurons has been a variation of single cell tracing, in which Golgi-like staining is achieved by dye injection. We believe that our novel vector system provides a useful option to this labor-intensive task. Below, we discuss the advantage and disadvantage of our approach that emerged through our work compared with other methods of morphological analyses. In addition, we discuss the potential contribution of our vector system in investigation of the cortical projection neuron subtypes.

### Methodological considerations: advantage and disadvantage of the retrograde TET-Off vector

One of the biggest advantages of our retrograde vector system over the dye-filling techniques is the simplicity of the method. By a single shot of the TET-Off vector, multiple cells of the same projection profiles are simultaneously labeled for fine morphological analyses. Classic retrograde tracers, such as HRP, have been previously used in a similar way to investigate the morphology of a defined set of projection neurons (e.g., [33]). Such tracers, however, were only able to label cell bodies and thick dendrites. In contrast, our TET-Off vector (as well as other viral-based retrograde tracers; see below) can label the neurons to very fine structure. Our vector, thus, provides a simple solution for the morphological analysis of cortical projection neurons. On the other hand, multiple labeling makes it very difficult to isolate a single cell out of co-labeled populations. We consider that our vector system is most suited to analyze the shared feature of a defined population, such as the layer-specific corticofugal neuron subtypes, which are known to extend stereotypical dendritic and axonal projections [2].

To date, there have been reports of retrograde tracers based on several different viral vectors, such as rabies [9], adenoviral vectors [34], [35], VSV [36] and herpes simplex virus [37], [38]. Among these, the glycoprotein deficient rabies vector [9] and adenoviral vector [34], [35] have been successfully used to analyze the morphology of particular projection neurons. The improvement that we incorporated in this study made the lentiviral-based vector system a useful option to achieve the same goal. There are, at least, two advantages of the lentiviral-based system.

First, the lentiviral-based system is considered relatively less toxic or inflammatory [39], and the transgene expression can be maintained for months. As we have shown in Fig. 3, lentiviral vectors potentially cause transient inflammatory responses at the injection site. However, after several weeks, the transduced cells showed no sign of damage based on stable NeuN expression, except in the injection center (see Fig. 8D and E). In contrast, the cells infected with rabies vector are reported to deteriorate after two weeks [9]. Adenoviral vector is also known to induce inflammatory responses and cytotoxic effects [40]. Second, in terms of practical handling, many levels of safety measures make lentiviral vectors relatively easy to handle. Although lentiviral vectors must be handled with biosafety level 2-containment, they can be easily inactivated by alcohol. The emergence of replication competent viruses by recombination during production is not known so far for lentiviral vectors [41]. For many laboratories that have experience in lentiviral vectors, introduction of our vector system would be easy.

Through the use of our retrograde TET-Off vector, however, we also noticed several disadvantages that must be improved in the further study. First of all, the local infection of the retrograde vector is quite problematic. The extensive labeling to the tips of axons confounds the morphological analyses in the regions with reciprocal connectivity, such as in the corticocortical connections. This is probably, more or less, a common problem to any viral-based retrograde tracers, because neurons generally have local axon collaterals. One way to circumvent this problem is to use the double infection strategy, which was recently developed [42]. Second, we do not think that the labeling by the TET-Off vector is saturated even after four weeks of infection. Depending on the cell type (e.g., small corticocortical neurons), the dendritic spines were only faintly labeled by RFP or palGFP (data not shown). In this regard, rabies-based vector replicates during infection, leading to very high transgene expression [9]. Third, the infectious titer of our retrograde TET-Off vector was not very high, even after concentration. This low titer should be one of the reasons for lower transduction efficiency of our TET-Off vector compared with CTB (Fig. 4). Depending on the applications, we would need to improve the construct and/or packaging vectors to obtain higher infection rate.

### Retrograde Tet-Off lentiviral vector is a useful tool to investigate the layer-specific elaboration of neurite extension

In this paper, we investigated the bulk morphology of corticothalamic, corticocortical and corticopontine cells by the viral strategy, which was overall consistent with the previous studies [22]–[24], [26], [43]. The strength of our data is that the bulk labeling of neurites was directly compared with the lamina markers (NeuN and Hoechst dye) for exact matching. For example, we found it quite impressive that many axon collaterals of the corticothalamic cells bend horizontally within layer 5, as if there were a “ceiling” of neurite extension (e.g., see Fig. 2D, Fig. 3K and M). We also observed that the dendrites of the corticopontine cells are strictly confined within layer 5 (Fig. 6I). Based on the developmental studies [44], [45], there probably exist both genetic and activity-dependent mechanisms to control layer-specific elaboration and maintenance of axons and dendrites. Nevertheless, surprisingly little is known about the actual mechanism that sculpts the above-mentioned laminar restrictions.

It is now well established that the cortical projection neuron subtypes are defined early during development by a cascade of gene regulatory events [1], [46], [47]. In the downstream of such events are the expressions of axon guidance molecules, adhesion molecules and other signaling molecules [48]. Our vector system will be useful to investigate the roles of these genes, by visualizing the morphology of particular projection neuron types as we did in this study.

Finally, we call attention to the potential importance of our vector in the primate study. One big difference that sets human apart from other mammals is the expansion of the cerebral cortex, caused by the increase in neuron number. This expansion is accompanied by a highly elaborated corticocortical network, which is investigated in detail for the macaque cortex [49], [50]. Based on gene expression and laminar connectivity in mouse, rat and macaque, the corticocortical neurons that support this complex network are considered to consist of heterogeneous subpopulations [26], [32], [51]–[54]. But the corticocortical neuron subtypes that connect various primate areas still need to be characterized. In this regard, retrograde adenoviral vectors and rabies vectors [35], [55] have been successfully used to investigate the corticocortical neurons in macaques. The lentiviral-based vector used in this study should be a useful option in investigating the primate cortical connections.
